# More Than One Disease Process in Chronic Sinusitis Based on Mucin Fragmentation Patterns and Amino Acid Analysis

**DOI:** 10.1155/2015/708475

**Published:** 2015-01-26

**Authors:** Mahmoud El-Sayed Ali, Jeffrey P. Pearson

**Affiliations:** ^1^Department of Otolaryngology, Mansoura University Hospital, Mansoura University, Mansoura 35516, Egypt; ^2^Institute for Cell and Molecular Biosciences, Faculty of Medical Sciences, Newcastle University, Newcastle upon Tyne NE2 4HH, UK

## Abstract

*Objective*. To characterise fragmentation patterns and amino acid composition of MUC2 and MUC5AC in chronic sinusitis.* Methods*. Antigenic identity of purified sinus mucins was determined by ELISA. Fragmentation patterns of a MUC5AC rich sample mucin were analysed by Sepharose CL-2B gel chromatography. Samples, divided into one MUC2 rich and one MUC5AC rich group, were subjected to sodium dodecyl sulphate-polyacrylamide gel electrophoresis (SDS-PAGE) and their amino acid contents were analysed.* Results*. Reduction, trypsin digestion, and papain digestion produced progressively smaller mucin species. On SDS-PAGE, digested MUC5AC rich mucin produced four distinct products. Amino acid analysis was characteristic of mucins with high serine, threonine, and proline contents and reduction and proteolysis increased relative proportions of these amino acids. MUC5AC rich mucins contained more protein than MUC2 rich mucins.* Conclusion*. Sinus mucin fragmentation produced mucin subunits and glycopeptide units of smaller molecular sizes which are likely to have lower viscoelastic properties. Applying this in vivo could alter mucus physical properties and biologic functions. Amino acid contents of MUC2 and MUC5AC mucins are different. This could be contributing to biological properties and functions of sinus mucins. These data suggest that there may be different pathological processes occurring at the cellular level on chronic sinusitis.

## 1. Introduction 

Mucin production is controlled by mucin genes (MUCs). To date, 21 human mucin genes (MUCs 1, 2, 3A, 3B, 4, 5AC, 5B, 6–9, 12, 13, and 15–22) have been identified by cDNA cloning [1–4] and all, excluding MUCs 9, 11, 16, and 17, are expressed in airway mucosa [3, 4]. Several studies have shown that the major mucins expressed in chronic sinusitis (CS) are MUC2, MUC5AC, and MUC5B [5–9] and an inverse relationship was found between MUC2 and MUC5AC expression levels and this was strong in the presence of nasal polyps [6].

Mucins represent the major constituent responsible for mucus viscoelastic properties [10] which enables the mucus layer to achieve its protective function for the underlying mucosa. Mucins are highly glycosylated protein molecules of linear and flexible amino acid polymers composed of subunits joined by disulphide bonds [11–13]. Each subunit contains highly glycosylated, proteinase-resistant regions (variable number tandem repeat, VNTR) rich in serine and/or threonine amino acids alternating with sparsely or nonglycosylated (naked), proteinase-sensitive regions [14–16].

As sinus mucus is made up from multiple mucin gene products, it is important to understand the structure and composition of sinus mucins and how their distribution varies in sinus mucus. Alteration of mucus quantity and/or quality could alter mucus viscoelastic properties leading to alterations of the mucociliary clearance. From the clinical point of view, this alteration could aggravate patients' symptoms of mucus rhinorrhea and difficulty in mucus clearance with the development of other nasal, pharyngeal, and laryngeal symptoms. From the pathopharmacological point of view, altered mucus physical and/or biological properties could affect the penetration of mucus by pathogenic and/or therapeutic agents. We studied the sinus mucin fragmentation as this will help in the understanding of the physical and biological properties of sinus mucins in CS and could help to develop therapeutic modalities to alter these properties and facilitate mucus drainage and relieve relevant CS symptoms such as rhinorrhea, chronic cough, and globus pharyngeus.

## 2. Methods

Sinus mucus was collected from 8 patients undergoing functional endoscopic sinus surgery. Chronic sinusitis is defined as persistence of sinus symptoms (nasal obstruction, discharge, reduced sense of smell, headache, and facial pain) for more than 3 months (with topical steroids used for at least 3 months) [17, 18].

The patients' group included 5 males and 3 females, aged from 19 to 78 years with mean age of 43 years. Mucus samples were collected from the maxillary sinuses through the middle meatal antrostomy (MMA) during functional endoscopic sinus surgery (FESS). Native/polymeric mucins were isolated by 2 rounds of caesium chloride density gradient centrifugation [19] followed by extensive dialysis and freeze-drying. The antigenic identity of sinus mucins was determined by an enzyme linked immunosorbent assay (ELISA) using antibodies for MUC2, MUC5AC, and MUC5B mucins as described elsewhere [6].

### 2.1. Fragmentation of MUC5AC and MUC2 Mucins

Polymeric sinus mucins were fragmented by reduction with dithiothreitol (DTT) or proteolytic digestion with trypsin or papain.

#### 2.1.1. Reduction

2 mg of polymeric mucin was incubated with 10 mM DTT, 20 mM tris/HCl, and 6 M guanidinium chloride, pH 8, 37°C, for 5 h. Solid iodoacetamide 25 mM was then added to block the free-SH groups and the incubation continued overnight at room temperature in the dark.

#### 2.1.2. Proteolytic Digestion

2 mg of mucin from purified sinus mucin sample (S1) rich in MUC5AC mucin was incubated with either porcine trypsin 0.5 *μ*g/mg of mucin in 100 mM ammonium hydrogen carbonate buffer pH 8.0 at 37°C for 24 h or papain 40 *μ*g/mg of mucin in 0.1 M sodium phosphate buffer containing 5 mM cysteine HCl, 10 mM EDTA, pH 6.25 at 60°C for 48 h.

After extensive dialysis, polymeric and fragmented mucins were fractionated by gel filtration chromatography using a Sepharose CL-2B column (125 × 1.5 cm) and eluted by upward flow with 0.2 M NaCl and 0.02% (w/v) NaN_3_ and 2 mL fractions were collected. Glycoprotein contents were measured by the periodic acid Schiff (PAS) assay. The Kav values were calculated for the glycoprotein rich peaks.

### 2.2. Sodium Dodecyl Sulphate-Polyacrylamide Gel Electrophoresis (SDS-PAGE)

After gel chromatography, polymeric and fragmented mucins were subjected to SDS-PAGE on 4–15% gradient gels. Mucin rich peaks from gel filtration were pooled and dialysed against distilled water for 48 h and then freeze-dried and 1 *μ*L aliquots of 5 mg/mL mucin solutions in nonreducing loading buffer were applied to the gels. After electrophoresis, gels were stained with PAS and scanned at 555 nm using a Shimadzu dual wavelength chromatoscanner. Sinus mucin samples rich in MUC5AC and MUC2 mucins were subjected to SDS-PAGE.

### 2.3. Amino Acid Analysis

This was performed by a modified version of the method of Carlton and Morgan [20] which allowed analysis of small amounts of mucins (~10 *μ*g). Norvaline was used as an internal standard and Sigma amino acid mixture was used as an external standard. Samples were vapour-phase-hydrolysed in 6 M HCl for 1 h at 165°C and derivatized with 9-fluorenylmethyl chloroformate prior to analysis with reverse phase high performance liquid chromatography (HPLC) [21]. Amino acid contents were expressed as means ± standard error of the mean (S.E.M.).

## 3. Results 

Four sinus mucin samples had MUC5AC mucin as the major mucin (S1–4) and the other four samples had MUC2 mucin as the major mucin (S5–8).

### 3.1. Gel Chromatography-Size Distribution of Mucins

**Figure 1:**
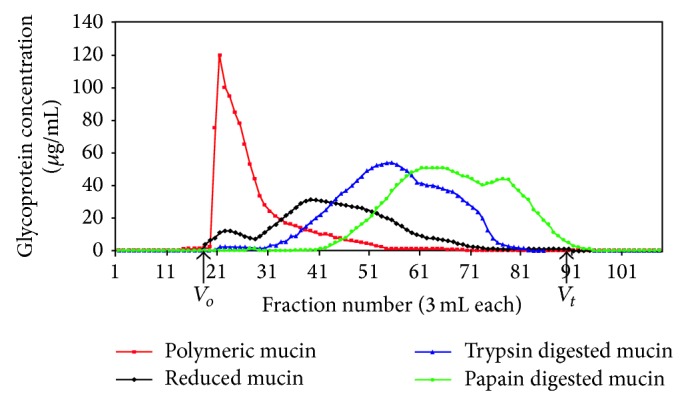
Chromatographic profile of the purified fractionated sinus mucin sample (S1). Sepharose CL-2B gel column (125 × 2.5 cm) was eluted by upward flow with 0.2 M sodium chloride containing 0.02% (w/v) sodium azide and flow rate 18 mL/h. Calibration was firstly performed using 1% (w/v) dextran blue solution containing 0.05% (w/v) methyl orange. The glycoprotein content was estimated by the PAS solution assay. *V*_*o*_ and *V*_*t*_ are the void and total volumes, respectively.

Gel filtration was performed on MUC5AC rich sinus mucin sample (S1). The elution profiles are shown in Figure 1. Seventy-five percent of polymeric sinus mucin was excluded (Kav 0) and the remaining 25% spread into the partially included volume as a trailing edge. On reduction, sinus mucin eluted as two peaks: the first, representing 20% of the mucin, was excluded and the other 80% eluted as a partially included broad second peak (Kav 0.28). Trypsin digested mucin eluted as two included peaks. First peak made up 80% of the mucin (Kav 0.47) and the second (20%) constituted a shoulder of smaller size material (Kav 0.69). Papain digested polymeric mucin showed two included, partially separated peaks with Kav 0.57 and 0.78 accounting for 74% and 26% of loaded mucin, respectively.

### 3.2. SDS-PAGE

Electrophoretic patterns of polymeric and fragmented sinus mucins reflected those noted after gel chromatography. Polymeric mucin remained in the stacking gel phase whereas fragmented mucin migrated into the running gel phase to a distance dependent on its hydrodynamic size with a progressive diminishing of size from reduced to digested conditions (results not shown). Furthermore, SDS-PAGE of a polymeric MUC5AC rich mucin sample without gel chromatography showed that 76% of mucin remained at the point of application and 24% migrated from the point of application compared to 80% excluded and 20% included on gel filtration (results not shown). This indicated that SDS-PAGE can be used as an alternative method to gel chromatography to study sinus mucin fragmentation patterns.

**Figure 2:**
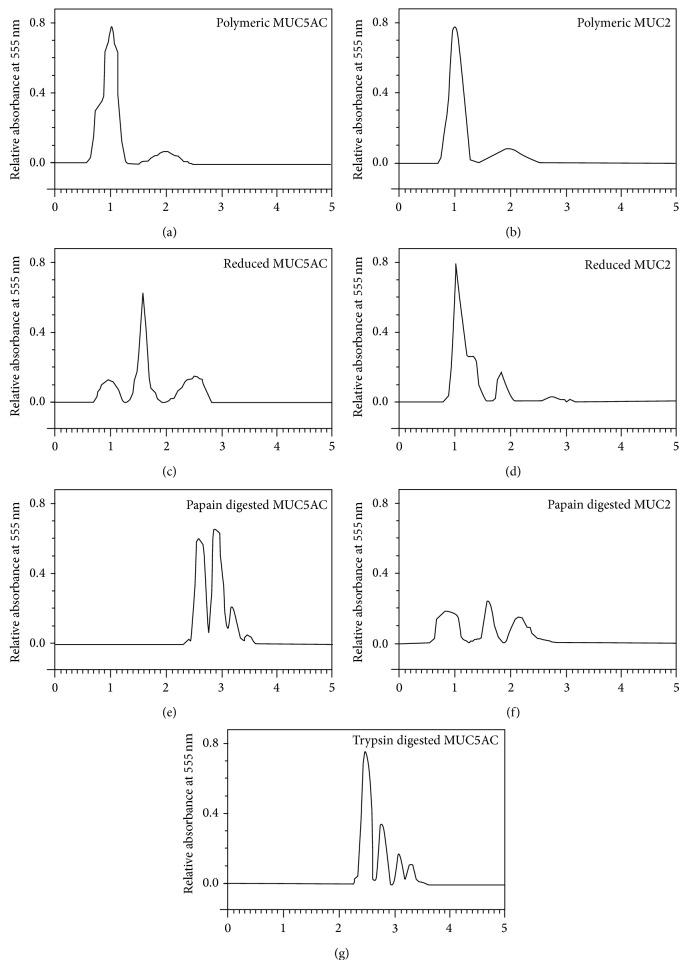
Densitometric scans of SDS-PAGE of polymeric and fragmented MUC5AC and MUC2 rich sinus mucins. The *x*-axis represents the localization of the different PAS-positive peaks relative to the site of application on the stacking gel (1) and the interface between the stacking and migration gel phases (2). The *y*-axis represents the relative absorbance of the different PAS-positive material (mucins) measured in arbitrary units. For the purpose of comparison of the different electrophoretic patterns, the presented figures were arranged as follows: ((a), (c), (e), and (g)) polymeric, reduced, papain digested, and trypsin digested MUC5AC rich mucins, respectively; ((b), (d), and (f)) polymeric, reduced, and papain digested MUC2 rich mucins, respectively.

Two sinus mucin samples, one rich in MUC5AC (S1) and one rich in MUC2 (S5) mucins, were subjected to SDS-PAGE. In the 2 samples, polymeric sinus mucins gave similar profiles, with the majority of the mucin at the point of application in the stacking gel and a small amount migrating to the interface between the stacking and running gels (Figures 2(a) and 2(b)). On reduction the two samples behaved differently. Twenty-four percent of PAS-staining material from MUC5AC rich mucin remained at the loading point and the rest migrated towards and into the running gel. Of this, 45% was located between the origin and the interface between the stacking and running gels and 30% spread from the interface up to 7.5 mm into the running gel (Figure 2(c)). In contrast, MUC2 rich mucin showed little evidence of any material in the running gel with 84% of PAS-staining material remaining at the origin and 16% migrating into the stacking gel (Figure 2(d)). Papain digested MUC5AC rich mucin produced four bands migrating 5.5, 8.5, 10.5, and 13.5 mm into the running gel and constituting 42%, 44%, 10%, and 4% of the total PAS-positive material, respectively (Figure 2(e)). MUC2 rich mucin produced a different profile with 42% of the glycoprotein remaining at the origin, 26% migrating into the stacking gel, and the remaining 32% forming a diffuse staining extending 10 mm in the running gel with no distinct bands (Figure 2(f)). Trypsin digested MUC5AC rich mucin produced four bands migrating into the running gel similar to those produced by papain digestion. However the distribution of staining was different as the second band was relatively smaller. The four bands made up 54%, 24%, 13%, and 9% of glycoprotein, respectively (Figure 2(g)). As papain digestion of the MUC2 rich samples did not give distinct running gel species, SDS-PAGE of trypsin digested MUC2 rich mucins was not performed.

### 3.3. Amino Acid Analysis of Human Sinus Mucin

Protein content of the mucins varied between 14.2% and 24.8% by weight. MUC5AC rich mucins had higher protein content than the MUC2 rich mucins, 21.1% ± 1.4% and 15.4% ± 0.8%, respectively. The general amino acid composition of sinus mucins showed the characteristic mucin analysis rich in serine, threonine, and proline. Total content of these 3 amino acids represented 29.7% to 49.6% by weight of the protein core. MUC5AC and MUC2 rich mucins contained total serine, threonine, and proline of 43.6% ± 3.7% and 40.1% ± 3.7%, respectively, the main difference being in serine content which was 7.8 ± 0.5% in MUC5AC rich mucins compared to 16.8 ± 2.1% for the MUC2 rich mucins (*P* = 0.02, paired *t*-test). Threonine represented 14% ± 0.6% in MUC5AC rich mucins compared to 12.1% ± 1.5% in the MUC2 rich mucins (insignificant difference). The mean serine/threonine ratio was 3 : 5 for MUC5AC rich mucins and 7 : 5 for MUC2 rich mucins. Aspartate and valine amino acids were present in higher levels in MUC5AC rich mucins (Table 1).

Following reduction, a seventh and a fifth of the protein content were lost for the MUC5AC and MUC2 rich mucins, respectively (Table 2). The reduced mucins still contained different amounts of protein (17.9 ± 1.3% and 12.0 ± 1.0% for MUC5AC and MUC2 rich mucins, resp.). The content of serine, threonine, and proline increased on reduction to 39.0 ± 1.6% and 42.5 ± 2.6% for MUC5AC and MUC2 rich mucins, respectively. Reduction did not release protein enriched in any particular amino acids.

Proteolytic digestion of sinus mucins produced a greater loss of protein than reduction with approximately one-third of the protein content lost. Digested mucins still had different protein contents which represented 14.4 ± 1.1% in MUC5AC rich mucin and 10.0 ± 0.9% in MUC2 rich mucin. The content of serine, threonine, and proline increased in both sinus mucin groups after proteolytic digestion to account for almost half the remaining protein, 48.8 ± 1.0% and 48.8 ± 2.3% for MUC5AC and MUC2 rich mucins, respectively. There was a large loss of acidic amino acids on proteolysis, with the MUC5AC rich mucins decreasing from 20% in the polymeric mucins to 8% in the digested mucins and the MUC2 rich mucins decreasing from 15% in the polymeric mucins to 9.5% in the digested mucins (Table 3).

## 4. Discussion 

This study does not include normal sinus mucin as a control. It would be more informative to have such a control to find out if there were differences in its antigenic identity, polymeric structure, and fragmentation behaviour compared to that in chronic sinusitis. There are ethical and technical difficulties to obtain normal sinus mucus for mucin extraction. This would entail an unnecessary invasive procedure to obtain maxillary sinus mucus from healthy sinuses. Furthermore, normal sinus mucus production is extremely small and it would not be possible to do the required biochemical analyses on such small amounts. This would then necessitate pooling different normal sinus mucus samples together to obtain adequate mucin material for these tests. This would not then allow the normal sinus mucin gene profile for each patient to be identified.

Mucins present in paranasal sinus mucus have been identified predominantly as MUC2, MUC5AC, and MUC5B [5, 6, 8, 9]. These are secretory mucins produced by surface epithelium goblet cells and submucosal glands [22, 23]. In this study, MUC5AC rich polymeric mucins were essentially excluded on Sepharose CL-2B giving similar pattern to that of colonic (MUC2 rich) and gastric (MUC5AC rich) mucins [21, 24, 25]. However, the 25% partially included component could represent smaller molecular weight mucin species or possibly products of in vivo mucin fragmentation/degradation and a function of the disease process and this indicates that chronic sinusitis mucin molecules are not of a homogenous size.

Due to limited available amount of mucin which remained after other mucin analyses including mucin identity studies (ELISA), the sinus mucin sample subjected to fragmentation and analysis on gel filtration was rich in MUC5AC. Not enough mucin was available to study gel chromatographic behavior of MUC2 rich samples. However, as the SDS-PAGE distribution of MUC5AC rich polymeric and fragmented mucins mirrored the chromatographic pattern of these mucins on Sepharose 2B gel filtration, SDS-PAGE allowed the study of mucin fragmentation in small quantities of mucin samples. This finding is similar to what was reported in other studies on gastric [25] and cervical [26] and respiratory [27] mucins.

Polymeric MUC5AC and MUC2 rich mucins are of large molecular size with the majority of the sample remaining at the point of application on the polyacrylamide gel. As the polymeric structure of MUC2 and MUC5AC mucin molecules is based on disulphide bridges, reduction produced a size change consistent with a subunit structure. However, MUC2 rich mucin showed little change compared to MUC5AC rich mucin. This is similar to previous finding in reduced pig colonic mucin, rich in MUC2, which was largely excluded on Sepharose Cl-2B gel chromatography [21].

Papain digestion of the MUC2 rich mucins did not produce distinct glycosylated units in the running gel whereas MUC5AC rich mucins produced four distinct species in the running gel after papain and trypsin digestion. The antigenic identity of these peaks was not determined but these could reflect different sized glycosylated domains in the MUC5AC molecule that are different in size and/or charge. It is unlikely that these bands represent MUC5B mucin as both the MUC2 and the MUC5AC rich samples contained MUC5B. Furthermore, it has also been reported that MUC5AC may be more susceptible to proteolytic degradation than MUC5B [13]. However the presence of other mucins, besides MUC5AC, that are not present in the MUC2 rich samples cannot be ruled out. These 4 bands could, provisionally, be considered as finger prints for sinus MUC5AC or MUC5AC rich sinus mucins.

The integrity of the polymeric structure is important for the gel-formation character of mucins. The polymeric structure of MUC2 and MUC5AC is based on disulphide bridges between mucin subunits and peptide bonds between mucin glycopeptides [1]. Therefore, disruption of these bonds by reduction or proteolytic digestion splits the mucin molecule polymer into subunits and glycopeptides, respectively, causing solubilisation of the mucus gel. In a pathophysiologic context, biologic agents such as bacteria could produce their pathogenic effect by altering the structure of mucin molecules in the mucus gel and lowering mucus viscosity [24, 25]. On the other hand, a therapeutic effect could be achieved by a mucolytic drug, such as N acetylcysteine, by lowering mucus viscosity [28]. Facilitating mucus clearance and/or penetration by therapeutic agents could contribute to the treatment of chronic airway infections.

Amino acid analysis of the polymeric mucins demonstrated that the two groups of mucins were rich in serine, threonine, and proline similar to reported levels of these amino acids in other well characterized polymeric mucins, that is, human gastric, cervical, respiratory, and middle ear [26, 29, 30]. There were however significant differences between the two mucin groups. MUC5AC rich mucins contained less serine than the MUC2 rich mucins. The proportion of serine/threonine ratio in MUC5AC samples was close to the reported ratio of these amino acids in MUC5AC molecule. However, this proportion was not as would be expected based on the consensus sequence for MUC2 gene and from the amino acid analysis of the insoluble glycoprotein complex from the human colon which consists of human MUC2 mucin where serine : threonine ratio was 1 : 4 [31]. Our mucins are from the sinus and not the colon and contain MUC5B and probably other mucins; that is, it is a mixed secretion and these facts could explain the lack of raised threonine relative to serine in the MUC2 rich samples.

On reduction nearly similar amounts of protein were lost in both groups which meant that the reduced mucin groups still contained different amounts of protein. This amount of protein lost on reduction has previously been shown for pig colonic mucin [21].

Digestion of human sinus mucins results in approximately one-third of the protein content being lost. The proportions of serine, threonine, and proline have increased to almost one-half of the protein core; similar increases have been demonstrated for human middle ear and gastric mucins [30, 32] and the large loss of acidic amino acids on proteolysis is similar to what has been reported with human gastric and cervical mucins [28].

Detailed characterization of amino acid composition of sinus mucins could help identify potential amino acid sites for reduction and proteolytic activity to break down the mucin molecule and hence reduce its viscosity and facilitate its mucociliary clearance.

## 5. Conclusion 

MUC2 and MUC5AC expressed in chronic sinusitis mucus behave differently on reduction and proteolytic digestion. Proteolytic digestion of MUC5AC rich mucins produced mucin glycopeptides which, on SDS-PAGE, produced 4 distinctive bands which could be used as finger prints for MUC5AC. These mucins have different amino acid composition which, however, is similar to the general amino acid composition profile of known mucins.

Further characterisation of sinus mucins in health and disease conditions could help in the understanding of biological importance of polymeric sinus mucin molecules and its subspecies and might help in the invention of medical treatment modalities for chronic sinusitis.

This study does not include normal sinus mucin as a control. It would be more informative to have such a control to identify the differences in its polymeric structure and fragmentation behaviour compared to that in chronic sinusitis. There are ethical and technical difficulties to obtain normal sinus mucus. Normal nasal mucus might represent an alternative for sinus mucin. However, we have found that nasal and sinus mucin expression are not similar in disease [5] and it is not known if they are similar in health.

## Figures and Tables

**Table 1:** Amino acid composition of native/polymeric chronic sinusitis mucins.

Amino acid	S1	S2	S3	S4	S5	S6	S7	S8
	MUC5AC rich samples				MUC2 rich samples			
	% content of amino acids				% content of amino acids			
His	5.7 ± 0.2	9.9 ± 0.1	6.3 ± 0.7	8.5 ± 0.4	11.0 ± 1.8	1.8 ± 0.4	3.1 ± 0.1	4.1 ± 0.1
Arg	14.9 ± 1.9	16.8 ± 1.8	11.5 ± 1.4	14.6 ± 1.0	6.6 ± 0.6	13.7 ± 1.6	11.2 ± 1.7	24.5 ± 0.5
Ser	14.7 ± 0.2	18.5 ± 1.3	21.0 ± 2.1	12.2 ± 1.3	16.7 ± 2.3	35.7 ± 2.7	27.4 ± 3.5	23.7 ± 0.5
Thr	25.2 ± 0.7	33.2 ± 3.2	35.8 ± 0.8	24.6 ± 0.5	12.1 ± 0.5	25.2 ± 1.1	17.4 ± 1.8	20.1 ± 1.1
Asp	32.3 ± 0.3	19.5 ± 0.3	22.8 ± 0.2	21.3 ± 1.2	12.5 ± 1.5	11.4 ± 0.4	7.3 ± 1.0	6.2 ± 0.2
Glu	23.9 ± 0.4	19.5 ± 1.3	23.5 ± 0.4	12.8 ± 0.9	20.0 ± 1.2	15.6 ± 1.4	7.8 ± 1.2	10.3 ± 0.8
Gly	12.4 ± 0.1	7.8 ± 0.1	13.3 ± 1.2	7.2 ± 0.2	10.3 ± 0.4	3.9 ± 0.9	6.8 ± 0.4	5.3 ± 0.7
Ala	18.1 ± 0.8	23.6 ± 0.6	25.0 ± 0.9	31.0 ± 3.4	12.2 ± 1.4	22.9 ± 2.7	18.0 ± 1.2	19.1 ± 0.1
Pro	21.2. ± 2.8	26.9 ± 1.9	32.3 ± 3.1	27.9 ± 0.1	17.7 ± 0.3	23.1 ± 1.6	15.4 ± 2.3	12.8 ± 0.8
Val	9.5 ± 0.5	12.3 ± 0.3	16.5 ± 0.4	9.7 ± 0.7	7.2 ± 0.7	7.0 ± 0.8	6.6 ± 0.4	3.0 ± 1.1
Ile/Leu/Phe	16.4 ± 0.2	16.6 ± 0.7	23.8 ± 0.1	18.2 ± 0.8	15.0 ± 1.8	11.2 ± 1.3	15.6 ± 1.7	9.7 ± 2.3
Lys	13.0 ± 1.3	11.9 ± 1.8	16.5 ± 1.4	5.0 ± 1.5	11.6 ± 1.5	3.9 ± 0.2	5.8 ± 1.2	4.1 ± 0.9
Calc. ptn	207.3 ± 1.1	216.5 ± 4.5	248.3 ± 5.6	193.0 ± 3.6	152.9 ± 8.3	175.4 ± 14.3	142.2 ± 8.2	142.9 ± 4.0

The values quoted are of the % amino acid contents in polymeric mucins. The samples were arranged according to the proportions of the relevant predominant mucin. In the first 4 samples MUC5AC (S1–S4) was predominant and MUC2 was predominant in the other 4 samples (S5–S8). Amino acid content was calculated in quadruplicate samples and the mean content of amino acid in each sample was calculated. Calc. ptn: calculated total protein in ug/mg of freeze-dried mucin.

**Table 2:** Amino acid composition of reduced chronic sinusitis mucins.

Amino acid	S1	S2	S3	S4	S5	S6	S7	S8
	MUC5AC rich samples				MUC2 rich samples			
	% content of amino acids				% content of amino acids			
His	3.8 ± .0.7	1.6 ± 0.4	2.1 ± 0.1	5.5 ± 0.4	7.8 ± 0.7	2.3 ± 0.3	3.5 ± 0.1	1.5 ± 0.3
Arg	20.4 ± 1.4	13.2 ± 1.7	7.2 ± 0.5	9.7 ± 0.7	4.2 ± 0.2	9.9 ± 1.0	5.9 ± 0.4	7.3 ± 0.7
Ser	27.9 ± 0.1	12.4 ± 1.3	22.5 ± 1.5	14.3 ± 0.7	11.1 ± 1.1	27.8 ± 0.8	25.5 ± 1.6	18.6 ± 1.6
Thr	30.6 ± 0.5	25.4 ± 2.4	32.9 ± 1.2	25.8 ± 2.1	21.2 ± 0.2	19.4 ± 1.2	17.8 ± 0.8	9.4 ± 0.4
Asp	32.4 ± 0.4	19.7 ± 1.6	30.5 ± 2.2	20.6 ± 0.6	11.2 ± 0.3	7.2 ± 0.2	4.9 ± 0.4	20.2 ± 1.6
Glu	9.6 ± 1.4	9.8 ± 0.2	21.2 ± 1.2	11.1 ± 1.0	17.2 ± 0.2	8.2 ± 1.3	8.3 ± 0.3	4.5 ± 0.6
Gly	5.7 ± 1.2	6.8 ± 0.4	17.0 ± 1.3	14.5 ± 0.8	3.4 ± 0.2	6.6 ± 0.6	7.0 ± 0.2	1.0 ± 0.1
Ala	25.2 ± 1.0	22.0 ± 2.2	24.2 ± 1.1	24.2 ± 1.9	5.8 ± 0.8	14.7 ± 1.2	15.4 ± 1.5	10.7 ± 0.1
Pro	26.5 ± 2.7	27.9 ± 0.9	24.8 ± 2.2	16.1 ± 1.7	9.5 ± .0.5	24.2 ± 1.2	11.3 ± 0.2	11.4 ± 0.3
Val	4.6 ± 0.6	3.4 ± 0.6	9.1 ± 0.1	3.7 ± 0.2	2.9 ± 0.3	5.3 ± 0.3	4.6 ± 0.8	1.6 ± 0.4
Ile/Leu/Phe	14.6 ± 1.8	0.9 ± 1.5	11.0 ± 1.1	13.0 ± 1.2	6.9 ± 0.3	15.1 ± 1.3	20.8 ± 0.8	13.7 ± 1.5
Lys	5.4 ± 1.0	5.5 ± 0.4	7.6 ± 1.1	2.6 ± 0.2	8.8 ± 0.2	2.9 ± 0.5	3.6 ± 0.7	00 ± 00
Calc. ptn	206.7 ± 10.2	156.7 ± 9.9	210.0 ± 13.4	161.1 ± 3.8	110.0 ± 2.2	143.6 ± 3.7	128.0 ± 3.4	99.7 ± 7.6

The values quoted are of the % amino acid contents in reduced sinus mucins. The samples were arranged according to the proportions of the relevant predominant mucin. In the first 4 samples MUC5AC (S1–S4) was predominant and MUC2 was predominant in the other 4 samples (S5–S8). Amino acid content was calculated in quadruplicate samples and the mean content of amino acid in each sample was calculated. Calc. ptn: calculated total protein in ug/mg of freeze-dried mucin.

**Table 3:** Amino acid composition of papain digested chronic sinusitis mucins.

Amino acid	S1	S2	S3	S4	S5	S6	S7	S8
	MUC5AC rich samples				MUC2 rich samples			
	% content of amino acids				% content of amino acids			
His	2.3 ± 0.2	7.8 ± 0.5	7.4 ± 1.5	2.4 ± 0.3	5.2 ± 1.4	0.0 ± 0.0	3.6 ± 0.1	1.1 ± 0.1
Arg	9.8 ± 0.3	8.4 ± 0.4	6.2 ± 0.7	6.6 ± 2.6	4.1 ± 0.1	7.5 ± 0.1	3.9 ± 0.4	8.3 ± 0.6
Ser	26.2 ± 0.2	32.8 ± 2.8	34.9 ± 1.9	17.2 ± 2.7	14.3 ± 2.0	30.3 ± 0.6	19.8 ± 0.1	22.6 ± 1.6
Thr	17.1 ± 0.5	19.6 ± 1.5	30.3 ± 0.5	16.9 ± 0.9	18.0 ± 0.9	20.3 ± 0.9	12.5 ± 0.5	14.1 ± 1.3
Asp	4.8 ± 0.3	2.8 ± 0.1	4.6 ± 0.6	6.6 ± 1.1	4.9 ± 0.2	3.1 ± 0.1	2.5 ± 0.4	3.1 ± 0.1
Glu	3.9 ± 0.8	3.6 ± 0.3	12.8 ± 0.2	5.6 ± 0.7	9.5 ± 0.2	3.0 ± 0.6	5.6 ± 0.9	4.4 ± 0.5
Gly	00 ± 00	0.0 ± 00	3.4 ± 1.3	0.1 ± 0.1	7.2 ± 0.1	2.2 ± 0.3	7.3 ± 0.3	4.7 ± 0.2
Ala	27.7 ± 2.5	19.6 ± 0.6	22.4 ± 1.4	18.4 ± 3.2	3.6 ± 0.3	28.4 ± 0.3	14.8 ± 1.9	10.3 ± 1.3
Pro	23.1 ± 1.1	18.2 ± 0.3	20.7 ± 1.9	24.1 ± 4.1	5.8 ± 2.0	17.5 ± 2.1	13.2 ± 0.8	8.5 ± 0.1
Val	3.1 ± 0.1	4.2 ± 0.7	4.6 ± 0.1	6.0 ± 1.0	1.9 ± 0.4	2.8 ± 0.1	1.9 ± 0.1	5.6 ± 0.2
Ile/Leu/Phe	23.00 ± 1.5	16.2 ± 1.0	17.8 ± 2.8	12.6 ± 1.0	7.1 ± 0.7	11.4 ± 1.4	4.5 ± 0.2	7.8 ± 0.9
Lys	2.8 ± 0.5	7.0 ± 0.4	6.0 ± 2.4	3.6 ± 0.9	7.4 ± 0.2	0.1 ± 0.1	1.5 ± 0.4	2.5 ± 0.2
Calc. ptn	143.8 ± 2.2	140.2 ± 2.6	171.1 ± 6.9	120.1 ± 0.5	89.0 ± 0.2	126.6 ± 3.3	91.1 ± 2.7	93.0 ± 1.0

The values quoted are of the % amino acid contents in papain digested sinus mucins. The samples were arranged according to the proportions of the relevant predominant mucin. In the first 4 samples MUC5AC (S1–S4) was predominant and MUC2 was predominant in the other 4 samples (S5–S8). Amino acid content was calculated in quadruplicate samples and the mean content of amino acid in each sample was calculated. Calc. ptn: calculated total protein in ug/mg of freeze-dried mucin.
